# Adiponectin inhibits oxidization-induced differentiation of T helper cells through inhibiting costimulatory CD40 and CD80

**DOI:** 10.1590/1414-431X20176227

**Published:** 2017-05-15

**Authors:** Y. Xiao, T. Deng, Z. Shang, D. Wang

**Affiliations:** Department of Hematology, Tongji Hospital, Tongji Medical College, Huazhong University of Science and Technology, Wuhan, China

**Keywords:** Adiponectin, T helper cells, HLA-DR, CD80, CD40

## Abstract

Adiponectin is a multifunctional adipokine that has several oligomeric forms in the blood stream, which broadly regulates innate and acquired immunity. Therefore, in this study, we aimed to observe the differentiation of T helper (Th) cells and expression of costimulatory signaling molecules affected by adiponectin. The mRNA and protein expression levels of adiponectin and its receptors in oxidized low density lipoprotein cholesterol-treated endothelial cells were assayed by real time PCR and immunofluorescence. The endothelial cells were then treated with adiponectin with or without adipoR1 or adipoR2 siRNA and co-cultured with T lymphocytes. The distribution of Th1, Th2 and Th17 subsets were assayed by flow cytometry. The effects of adiponectin on costimulatory signaling molecules HLA-DR, CD80, CD86 and CD 40 was also assayed by flow cytometry. The results showed that endothelial cells expressed adiponectin and its receptor adipoR1 and adipoR2, but not T-cadherin. Adiponectin suppressed Th1 and Th17 differentiation through adipoR1 receptor, contributed to the inhibition of CD80 and CD40, and inhibited differentiation of Th1 and Th17 by inhibiting antigen presenting action.

## Introduction

Adipose tissues can secrete a variety of adipokines such as leptin, resistin, adiponectin, plasminogen activator inhibitor-1, which participate in the regulation of a number of chronic diseases such as diabetes, cardiovascular diseases and cancers (1–4). Adiponectin is recognized as an important protector to the above diseases and it is usually markedly reduced in many kinds of metabolic diseases (5). It is a protein hormone produced by adipose tissues and released into the blood stream at a concentration of approximately 0.5-30 μg/mL, which is 1,000-fold higher than most other hormones (6). The effects of adiponectin in the regulation of insulin sensitivity have been documented since 1996 (7,8). Recently, adiponectin has also been shown to play a role in the protection and inhibition of cardiovascular diseases (9,10).

Growing evidence shows that atherosclerosis (AS) is a chronic inflammatory disease in which autoimmunity may play an important role. T lymphocytes are important immune cells participating in AS. An increase in the total number of T lymphocytes and activation of T lymphocytes in peripheral blood can be detected in hyperlipidemia and unstable angina patients (11). Also, a large number of T lymphocytes, especially CD4^+^ T lymphocytes, are found in atherosclerotic plaque in humans and rat models (12). Again, T lymphocytes can react with related antigen of artery atheromatous plaque, such as oxidized low density lipoprotein cholesterol (ox-LDL) (13), heat shock protein (14), etc., so as to promote the formation of AS. In addition, the inhibition of Th1 or Th17 pathways also reduces atherosclerotic lesions (15).

The activation of naive CD4^+^ T lymphocytes requires two signals delivered by antigen presenting cells: first, the interaction of specific major histocompatibility complex-peptide with the T-cell receptor, and second, a costimulatory signal. Among the costimulatory molecules, CD80 and CD86 are the best characterized. The CD40/CD40L system is also a crucial costimulatory molecule involved in differentiation of Th1 and Th17 (16,17). Some reports show that shutting down the CD40/CD40L system is very effective in dampening inflammation in *in vitro* cellular systems as well as *in vivo* animal models of experimental AS (18,19). Many signalings were coupled to CD40; for example, P38 MAPK/TNFα and PI3K/Akt /NF-kappab have been shown to enlarge systemic inflammation and promote differentiation of Th1 and Th17 (20).

Here, we aimed to observe the effect of adiponectin on the differentiation of T helper (Th) cells and expression of costimulatory signaling molecules in T lymphocytes treated with ox-LDL.

## Material and Methods

### Ethics statement

Peripheral blood was drawn from healthy adult volunteers. Before the experiments, we obtained approval for our study from the Ethics Committee of Tongji Hospital, Tongji Medical College, Huazhong University of Science and Technology and written informed consent from all study participants.

### Separation and culture of human umbilical vein endothelial cells (HUVEC)

Newborn umbilical cords were obtained under aseptic condition and washed in cold PBS. Then, the umbilical veins were injected with 10 mL collagenase (1 mg/mL) for digestion of endothelial cells at room temperature for 30 minutes. Digested cells were collected into a 50 mL sterile centrifuge tube by washing twice with sterile PBS at 1000 *g* at room temperature for 10 min and then cultured in DMEM medium supplemented with 15% fetal bovine serum at 37°C in 5% CO_2_. The culture media was replaced by fresh media every 2 days.

### Real-time PCR

The total RNA was isolated from HUVEC cells by using TRIzol¯ Reagent (Thermofisher Scientific, USA). Then, RNA was reverse transcribed to cDNA using PrimeScript™ RT reagent Kit (Takara, China) and analyzed using a Mini8 Plus Detection System (Coyote, USA). Real-time PCR was performed in a total of 20 µL reaction volume containing 1 µL of cDNA, 0.6 µL each of forward- and reverse-specific primers (from 25 pmol/µL primer stock) and Sybr green I. All qRT-PCR reactions were performed using the following reaction conditions: initial denaturation at 94°C for 4 min followed by 35 cycles at 94°C for 30 s, 60°C for 30 s, and then 72°C extension for 10 min. The primers of adiponectin were 5′-TATTGGTCCTAAGGGAGACATCGC-3′ and 5′-TTTTGGTGATACTACCGAGGTGAC-3′; for adipoR1 5′-CTGACTGGCTAAAGGACAACGACTA-3′ and 5′-TCGTAGAAGGCGTAAGTATGTCTT-3′; for adipoR2 5′-CGAAACCCGTCATTCAGCAGT-3′ and 5′-TTACCTTCCGACCGAACCTAC-3′; for T-cadherin 5′-TGTGGGTTAGTATTGGTGTATGTATGAGT-3′ and 5′-TTTGATTCTGTGGACTTGGGAGGTC-3′. Target genes were normalized to β-actin and quantified using 2^-ΔΔCt^ method (21).

### Immunofluorescence

The HUVEC cells were fixed, permeabilized and subsequently combined with rabbit anti-adipoR1, anti-adipoR2 or anti-T-cadherin antibodies from Abcam (UK). Then, the cells were incubated with FITC-conjugated anti-IgG antibodies (Sigma, USA) in the dark. The cells were mounted and observed on a fluorescence microscope (Olympus, USA).

### Separation of peripheral lymphocytes

Heparinized whole blood was collected from healthy adult volunteers. Peripheral blood mononuclear cells (PBMCs) were isolated by Ficoll-Paque plus (Absin, China) density gradient centrifugation. Then, PBMCs were incubated in complete medium at 37°C in 5% CO_2_ for 2 h until the monocytes adhered to the bottom of culture flasks. Then the lymphocytes suspended in medium were isolated.

After, PBMCs were incubated in complete medium at 37°C in 5% CO_2_ for 2 h until the monocytes adhered to the bottom of culture flasks. The lymphocytes suspended in medium were isolated using a CD4^+^T Cell Isolation Kit (Miltenyi Biotec, Germany) by positive selection of CD45RA+CD4^+^ T cells and negative selection of CD45RO^+^CD4^+^ T cells. Cells were cultivated at 37°C in 10% human AB plasma-containing RPMI 1640 medium, supplemented with penicillin-streptomycin. A total of 5×10^5^ CD45RA^+^CD4^+^ T cells were cultured in 96-well plates along with 1×10^5^ beads coated with 5 μg/mL anti-CD3, anti-CD28 according to the manufacturer's instructions.

### Co-culture of HUVECs and lymphocytes

In co-cultured experiments, HUVECs (2×10^5^ cells/well) were treated with or without 10 μg/mL ox-LDL (Yuanyuan Biotechnology, China) for 4 h after pretreatment with 5 μg/mL adiponectin (Glycosilated Polypeptide, Prospec, Israel) for 2 h. Then, the medium was discarded and lymphocytes were added to the HUVECs at a ratio of 1:1 so that HUVECs and lymphocytes were cultured in the same well. After incubation at 37°C in 5% CO_2_ for 48 h, the lymphocytes were collected.

### Flow cytometric analysis of Th subtypes

Lymphocytes were fixed with 4% paraformaldehyde for 10 min at room temperature and permeabilized in permeabilizing solution (eBioscience, USA). After blocking with 5% BSA for 20 min, cells were stained with appropriate anti-IL-4, IL-17 and IFN-gamma antibodies (Abcam, UK) on ice for 1 h. Isotype-matched antibodies were used as controls. The expression levels of antigens are reported as a percentage of positive cells in total cells.

### Flow cytometric analysis of costimulatory molecules

To assess the effect of adiponectin on costimulatory molecules, HUVECs were incubated with 10 μg/mL ox-LDL for 4 h after pretreatment with adiponectin for 2 h. Then they were washed twice with fresh media and treated with the monoclonal anti-human HLA, CD80, CD86 and CD40 (Biolegend, CA, USA). For each analysis, 10^5^ cells were incubated with 10 µL of monoclonal antibody at room temperature for 20 min. The cells were then washed twice and re-suspended in PBS containing 1% FBS and 0.1% NaN_3_ (Sigma) and immediately analyzed with FACS (Becton Dickinson, USA). Isotype-matched antibodies were used as controls. The levels of antigen expression are reported as a percentage of positive cells in the total cells.

### RNA interference

siRNAs Lentivector against AdipoR1 and AdipoR2, or control siRNA were obtained from Abm, Inc. (Canada). Endothelial cells were infected with control siRNA lentivirus, AdipoR1 siRNA or AdipoR2 siRNA with a multiplicity of infection of 1:10. After 48 h, transfected endothelial cells were incubated with 10 μg/mL ox-LDL for 4 h after pretreatment with adiponectin for 2 h. Control experiments have revealed that AdipoR1 and AdipoR2 mRNA levels are reduced by >80%.

### Statistical analysis

Data are reported as means±SE. The two groups were compared using the paired *t*-test. A two-way ANOVA was used to analyze Th subset distribution. A P value of <0.05 was considered to be statistically significant. Statistics were calculated using SPSS/Windows version 15.0.

## Results

### Endothelin cells expressed adiponectin and its receptors

**Figure 1.**
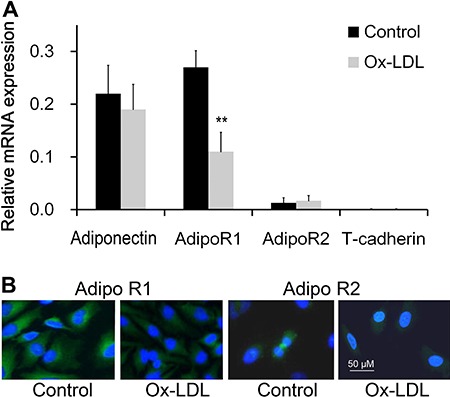
mRNA expression of adiponectin and its receptors in endothelial cells (n=4) assayed by real-time PCR (*A*). The mRNA expression level of adipoR1 decreased significantly at 8 h after treatment of oxidized low density lipoprotein cholesterol (ox-LDL). *B*, Adiponectin receptors identified by immunofluorescence (×400). **P<0.01 compared to control (paired *t*-test).

By using real-time PCR, we found that endothelial cells express the mRNA of adiponectin, adipoR1 and adipoR2, but not T-cadherin. The expression of adipoR1 was greater than that of adipoR2. After stimulation of 10 μg/mL ox-LDL, mRNA expression of adipoR1 decreased significantly at 8 h after treatment, while adipoR2 was not affected (Figure 1A). Immunofluorescence staining showed that adipoR1 and adipoR2 proteins were expressed in endothelial cells and adipoR1 decreased significantly at 8 h after oxidation stress (Figure 1B).

### Adiponectin rescued the decrease of adipoR1 induced by ox-LDL.

**Figure 2.**
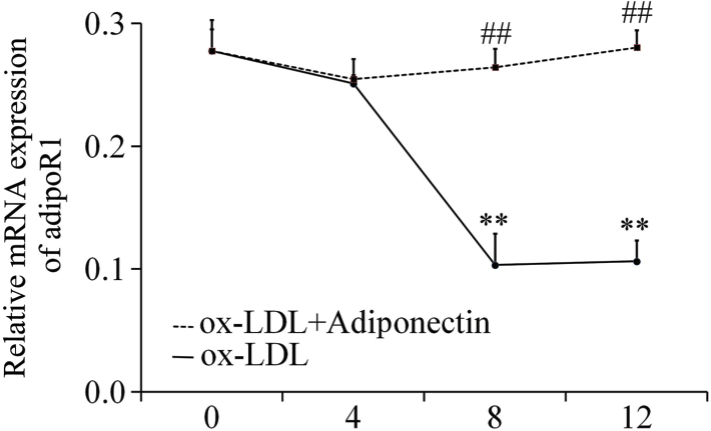
The effect of adiponectin on regulation of adipoR1 expression in cells treated with oxidized low density lipoprotein cholesterol (ox-LDL) was assayed by real-time PCR (n=4). AdipoR1 expression decreased with time and adiponectin rescued the decrease. **P<0.01 compared to time point 0 of ox-LDL; ^# #^P<0.01 compared to ox-LDL (paired *t*-test).

To investigate whether adiponectin acted through regulating its receptor adipoR1 expression, we stimulated the endothelial cells with 10 μg/mL ox-LDL for 4 h and assessed adipoR1 at 0, 4, 8, and 12 h later. The expression of adipoR1 significantly decreased after ox-LDL treatment, particularly at 8 and 12 h. With 5 μg/mL adiponectin co-incubation, the decrease of adipoR1 was rescued from 8 h after ox-LDL treatment (Figure 2).

### Adiponectin inhibited the differentiation of Th1 and Th17

**Figure 3.**
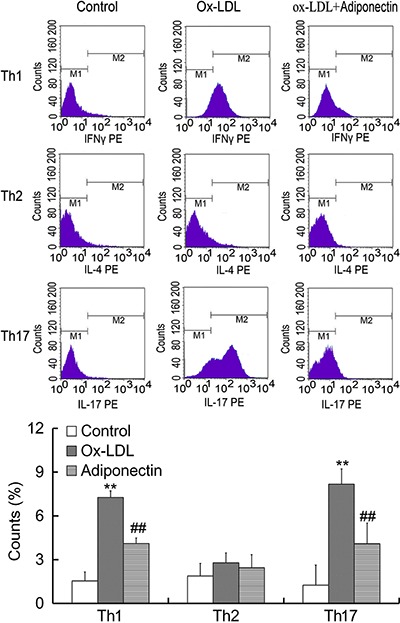
The differentiation of Th1, Th2 and Th17 in lymphocytes was assayed by flow cytometry (n=4). After treatment with adiponectin, the differentiation of Th1 and Th17 in lymphocytes induced by oxidized low density lipoprotein cholesterol (ox-LDL) was reduced compared with control. **P<0.01 *vs* control; ^##^P<0.01 *vs* ox-LDL (two-way ANOVA).

**Figure 4.**
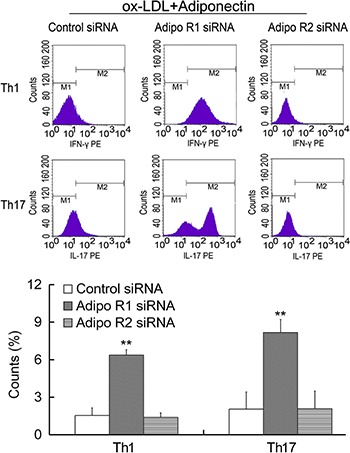
Inhibition of adiponectin on lymphocytes was reversed by adipoR1 siRNA (n=4). Endothelial cells were infected with control siRNA lentivirus, adipoR1 siRNA or adipoR2 siRNA with a MOI of 1:10. After 48 h, transfected endothelial cells were incubated with 10 μg/mL oxidized low density lipoprotein cholesterol (ox-LDL) for 4 h and were after pretreated with adiponectin for 2 h. Then, the medium was discarded and lymphocytes were added to the human umbilical vein endothelial cells (HUVEC) cells for 48 h, the lymphocytes were collected for analysis. **P<0.01 *vs* control (two-way ANOVA).

Th1 cells mainly secrete IFN-gamma, Th2 cells mainly secrete IL-4 and Th17 cells mainly secrete IL-17. Monensin (20 µL, 10 mg/mL) was used to inhibit the secretion of newly produced cytokines in Golgi body of lymphocytes. After intracellular staining, the distribution of Th1, Th2 and Th17 subsets were examined with flow cytometry. The results suggested that Th1 and Th17 differentiation induced by oxidation stress were suppressed with adiponectin treatment (Figure 3). Moreover, the inhibitory effects were abrogated by the treatment of adipoR1 siRNA (Figure 4), indicating that the inhibitory effects of adiponectin were mediated by adipoR1.

### Adiponectin inhibited the expression of CD80 and CD40

**Figure 5.**
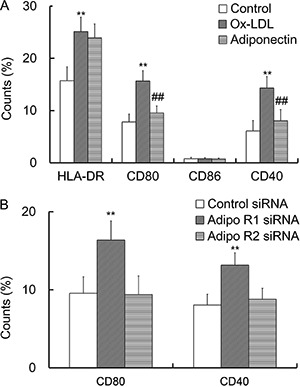
The expression levels of costimulatory molecules were assayed by flow cytometry (n=4). *A*, The expression levels of costimulatory molecules. **P<0.01 *vs* control; ^##^P<0.01 *vs* oxidized low density lipoprotein cholesterol (ox-LDL). *B*, The inhibition of adiponectin was reversed by adipoR1 siRNA. **P<0.01 *vs* control siRNA.

Constitutive expression of HLA, CD80, CD86 and CD40 molecules was observed on endothelial cells. Among them, HLA, CD80 and CD40 were up-regulated in the presence of ox-LDL, and adiponectin inhibited the expression of CD80 and CD40 induced by ox-LDL (Figure 5A). Moreover, the inhibitory effects were abrogated by the treatment of adipoR1 siRNA (Figure 5B).

## Discussion

Several studies have shown that adiponectin can reduce the area of lipid plaque, and inhibit neointimal thickening, smooth muscle cell proliferation and migration, and the expression of adhesion molecules on endothelial cells (22). It is reported that adiponectin suppresses TNF alpha-induced expression of adhesion molecules and mitogen activated protein (MAP) pathway-induced smooth muscle cell proliferation. In macrophages, adiponectin inhibits the expression of scavenger receptor and the formation of foam cells (23). Therefore, accumulation of adiponectin at the site of the lesions of endothelial cells plays a protective effect against AS. Currently, there are many reports about the relationship between adiponectin and AS. However, the specific roles of adiponectin in the regulation of immune responses remain unknown.

Using oxidized-injured endothelial cells, we aimed to determine whether and how adiponectin is involved in the immunological response. Therefore, we first tested the mRNA expression of adiponectin and its receptors in endothelial cells. We verified that endothelial cells express adiponectin and its receptor adipoR1 and adipoR2, but not T-cadherin. Ox-LDL can inhibit the expression of adipoR1, which can be rescued by additional administration of adiponectin. Next, the endothelial cells were treated with adiponectin with or without adipoR1 or adipoR2 siRNA, and then co-cultured with T lymphocytes. The distribution of Th1, Th2 and Th17 subsets were observed. The results showed that ox-LDL induced the differentiation of Th1 and Th17, and adiponectin suppressed Th1 and Th17 differentiation through adipoR1 receptor.

Among the accessory molecules involved in the activation of naïve CD4^+^ T lymphocytes , the up-regulation of CD40 is of particular interest in AS because the CD40 pathway is intimately involved in exaggerated inflammation (24). Indeed, stimulation of CD40-bearing cells triggers multiple inflammatory signals, resulting in leukocyte recruitment and amplification of tissue injury.

In conclusion, we found that endothelial cells were mediated in the abnormal differentiation of Th subsets, and adiponectin inhibited the differentiation of Th1 and Th17 subsets induced by ox-LDL through inhibiting CD80 and CD40 molecules.
